# Ivabradine in Septic Shock: A Narrative Review

**DOI:** 10.3390/jcm13082338

**Published:** 2024-04-18

**Authors:** Marco Pasetto, Lorenzo Antonino Calabrò, Filippo Annoni, Sabino Scolletta, Vincent Labbé, Katia Donadello, Fabio Silvio Taccone

**Affiliations:** 1 Department of Intensive Care, Hôpital Erasme, Université Libre de Bruxelles, 1070 Brussels, Belgium; 2 Department of Surgery, Dentistry, Gynecology and Paediatrics, University of Verona, 37129 Verona, Italy; 3 Anesthesia and Intensive Care Unit, Department of Medicine, Surgery and Neuroscience, University Hospital of Siena, 53100 Siena, Italy; 4 Anesthesia and Intensive Care Unit B, University Hospital Integrated Trust of Verona, 37134 Verona, Italy

**Keywords:** sepsis, septic shock, ivabradine, multiple organ disfunction syndrome, MODS, cardiac dysfunction, septic cardiomyopathy, β-blockers, ventriculo-arterial coupling

## Abstract

In patients with septic shock, compensatory tachycardia initially serves to maintain adequate cardiac output and tissue oxygenation but may persist despite appropriate fluid and vasopressor resuscitation. This sustained elevation in heart rate and altered heart rate variability, indicative of autonomic dysfunction, is a well-established independent predictor of adverse outcomes in critical illness. Elevated heart rate exacerbates myocardial oxygen demand, reduces ventricular filling time, compromises coronary perfusion during diastole, and impairs the isovolumetric relaxation phase of the cardiac cycle, contributing to ventricular-arterial decoupling. This also leads to increased ventricular and atrial filling pressures, with a heightened risk of arrhythmias. Ivabradine, a highly selective inhibitor of the sinoatrial node’s pacemaker current (I*_f_* or “funny” current), mitigates heart rate by modulating diastolic depolarization slope without affecting contractility. By exerting a selective chronotropic effect devoid of negative inotropic properties, ivabradine shows potential for improving hemodynamics in septic shock patients with cardiac dysfunction. This review evaluates the plausible mechanisms and existing evidence regarding the utility of ivabradine in managing patients with septic shock.

## 1. Introduction

In the early stages of septic shock, a life-threatening condition resulting from a dysregulated host response to infection that leads to multiple organ dysfunction syndrome (MODS) [1], compensatory tachycardia often occurs as a response to maintain adequate cardiac output and oxygen delivery to tissues [2,3]. This tachycardia aims to compensate for the acute reduction in preload and afterload, caused by vasodilation and relative hypovolemia. However, a significant number of patients develop persistent and refractory tachycardia even after receiving adequate treatment for distributive shock through fluids and vasopressors [4,5,6,7]. This phenomenon is often observed within the so-called sepsis-induced cardiac dysfunction, or septic cardiomyopathy [8,9,10].

Sepsis-induced cardiomyopathy exacerbates the imbalance between tissue oxygen supply and demand (DO_2_/VO_2_) during shock, worsening tissue hypoxia and contributing to the progression toward MODS. Moreover, with tachycardia, a ventricular-arterial decoupling may occur, leading to alterations of cardiovascular efficiency and cardiac energetic requirements [11,12,13,14,15]. Therefore, improving hemodynamics and cardiac function becomes one of the therapeutic priorities in the management of septic shock patients, following anti-infectious therapy and source control [2]. Ivabradine, a selective inhibitor of the sinoatrial node’s pacemaker current, modulates heart rate by reducing the diastolic depolarization slope [16]. Although the use of ivabradine to control tachycardia in patients with chronic heart failure has been studied [17,18], its effects on critically ill patients, particularly those with sepsis, remain to be elucidated.

This review aims at describing the existing literature on ivabradine’s potential application in septic shock and sepsis-induced cardiomyopathy, as well as discussing perspectives and potential pitfalls in clinical practice.

## 2. Hemodynamic Changes in Sepsis

The altered chronotropic response to distributive septic shock [19], which is no longer compensatory, results from excessive sympathetic stimulation originating from endogenous and exogenous sources [20,21,22,23]. These sources include fever, hypovolemia, endogenous and therapeutically applied catecholamines, inadequate sedation and/or pain control, and systemic and myocardial inflammation. This leads to cardiac autonomic dysfunction, which is characterized by a dominant sympathetic tone and depressed parasympathetic afferents. Autonomic dysfunction is not only reflected in the excessively high heart rate (HR) but also in narrowed heart rate variability (HRV), which represents the integrity of the autonomic nervous system [24,25,26,27]. Additionally, bacterial endotoxins and inflammatory mediators may directly affect the pacemaker activity of the sinoatrial (SA) node [28,29].

An excessively high HR and reduced HRV are associated with negative prognostic implications in MODS and increase the incidence of major cardiac events in critically ill patients [5,20,30,31,32,33]. In particular, high HR leads to several adverse effects, including increased myocardial oxygen consumption (MVO_2_), shortened myocardial perfusion time during diastole (particularly in the left coronary artery), prolonged systolic phases, exposing the heart to atherogenic flow and coronary shear stress (with an elevated risk of plaque rupture in coronary heart disease), a reduced relaxation phase of the cardiac cycle, leading to increased left and right ventricular diastolic pressures (LVEDP, RVEDP), increased left and right atrial pressures (LAP, RAP), and heightened risk of atrial fibrillation [20,34]. Furthermore, excessive sympathetic activity promotes automaticity and shortens the atrial effective refractory period, increasing the risk of atrial arrhythmias. High heart rates can also increase the risk of ventricular arrhythmias and exacerbate the “myocardial transmural steal” phenomenon in coronary patients, where blood flow decreases in stenotic vessels due to the coronary vasodilation in normal myocardium induced by tachycardia [20]. Prolonged sympathetic hyperactivity can disrupt myocardial contractility by causing a switch in adrenergic G protein coupling, shifting from a stimulatory to an inhibitory response to adrenergic stimulation, similar to the mechanism observed in Takotsubo cardiomyopathy [9].

Given that an excessively high HR and reduced HRV, indicative of autonomic dysfunction in MODS, are associated with unfavorable outcomes, interventions aimed at improving these variables may potentially enhance the prognosis of septic patients with MODS. Several studies have demonstrated that β1-selective blockers such as esmolol and landiolol effectively reduce heart rate, leading to improved ventricular-arterial coupling and enhanced hemodynamics [35,36,37,38,39]. Furthermore, β-blockers have shown efficacy in attenuating sympathetic autonomic dysfunction, restoring HRV, and limiting the harmful effects of sustained adrenergic stimulation, including myocyte necrosis, apoptosis, and increased arrhythmia risk [40,41,42,43,44]. Importantly, β-blockers have also positively impacted microcirculation in septic patients by modulating coagulation, metabolism, and inflammation [17,45,46].

However, concerns about their potential compromise of the already altered hemodynamics in septic shock patients who require catecholamines have limited the widespread use of β-blockers, especially when sepsis-induced cardiac dysfunction is suspected. Although β-blockers are effective in reducing HR, they also have a negative inotropic effect and thus may be potentially harmful in MODS patients with myocardial dysfunction [3,5]. These concerns are even supported by the premature interruption of the recent STRESS-L randomized trial using landiolol, a highly β1-selective blocker, for an increase in mortality and severity of organ dysfunction at 28 days associated with the use of this drug [47]. Similar issues emerge from the multi-center randomized J-Land 3S trial [48], where adverse events occurred in 64% of patients in the landiolol group, with serious adverse events, including events leading to death, in 12% of patients.

An alternative approach to reducing heart rate without inotropic compromise involves targeting the spontaneous depolarization of the I*_f_* (funny) current, responsible for SA node pacemaker activity. This can be achieved using the specific blocker of I*_f_* current channels, HCN-4 (hyperpolarization-activated cyclic-nucleotide 4), ivabradine.

## 3. Ivabradine Pharmacology

**Figure 1:**
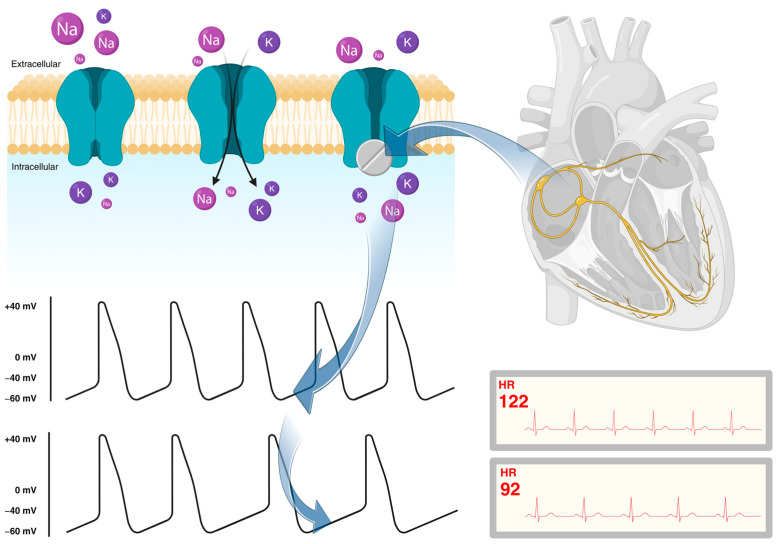
Ivabradine action on HCN-4 channels responsible for I*_f_* current, prolonging the diastolic slow depolarization phase of SAN myocytes. HCN = hyperpolarization-activated cyclic-nucleotide; Na = sodium; K = potassium; HR = heart rate.

Ivabradine functions as an antagonist for the transmembrane hyperpolarization-activated cyclic-nucleotide (HCN) gated channel, which exists in four isoforms in mammals. Among these isoforms, HCN-4 is predominantly found in SA node cells, where it governs the “funny” current (I*_f_*), determining the automaticity of the sinus node. The myocytes within the sinoatrial node exhibit a characteristic slow diastolic depolarization phase in their action potential, which is responsible for generating spontaneous activity and repetitive action potentials. I*_f_* current plays a crucial role during this depolarization phase by allowing the inward flow of Na^+^ and K^+^ ions through the HCN channels. These channels become activated at the end of the action potential when the membrane potential reaches approximately −40 to −50 mV. The amplitude of this current determines the slope of the diastolic depolarization phase, which, in turn, impacts heart rate (Figure 1) [17,18,49,50].

I*_f_* control over automaticity at the SA node is mediated by cyclic adenosine monophosphate (cAMP): A decrease in cAMP, induced by the release of acetylcholine (aCh), reduces the slope of depolarization of I*_f_*, resulting in a decrease in HR, while increased cAMP levels due to beta-adrenergic stimulation have the opposite effect [16,50,51,52,53]. Ivabradine reduces I*_f_* conductance by selectively binding to the HCN channel in its open state, resulting in use-dependent blockage. This leads to a more significant HR reduction at higher heart rates and occurs in a dose-dependent manner. Ivabradine specifically and selectively targets the I*_f_* current, without influencing intracardiac conduction, contractility, or ventricular repolarization. It does not interact with other myocyte channels such as T and L-type calcium channels, responsible for inotropy, or IK1/IK2 channels (controlling the duration of action potential) [17,51,54].

### 3.1. Ivabradine Pharmacodynamic/Pharmacokinetic

Upon enteral administration, peak plasma concentrations are reached within 1 h in the fasting state (2 h when fed), with a 40% oral bioavailability (increased by 20–40% in the fed state). Ivabradine is 70% protein-bound, with around 100 L volume of distribution at steady state; it also has a distribution half-life of 2 h, an elimination half-life of 6 h, resulting in an effective half-life of 11 h for drug efficacy. The drug is extensively metabolized via cytochrome P450 3A4 (CYP3A4) in the liver and intestines. Metabolites are excreted equally through feces and urine, while about 4% of an oral dose is excreted unchanged in the urine. Dose adjustments are not required for mild or moderate liver or renal impairment, but it is contraindicated in patients with severe hepatic dysfunction leading to drug accumulation. Caution is required with a creatinine clearance < 15 mL/min. Serum drug level monitoring is not necessary [55].

Ivabradine, as a weak competitive inhibitor of CYP3A4, does not impact the pharmacokinetics of other CYP3A4 substrates. However, its own pharmacokinetics can be modified by potent CYP3A4 inhibitors such as azole antifungals (e.g., ketoconazole, itraconazole), macrolide antibiotics (e.g., erythromycin, clarithromycin, josamicine, azithromycin, fidaxomicin, and telithromycin), HIV-protease inhibitors (e.g., nelfinavir, ritonavir), and nefazodone, which should not be co-administered with ivabradine. Additionally, moderate CYP3A4 inhibitors (such as diltiazem and verapamil) can exacerbate the adverse effects of ivabradine. CYP3A4 inducers (e.g., rifampicin, barbiturates, phenytoin) can reduce ivabradine plasma concentrations and, consequently, its activity [17,18,54].

### 3.2. Contraindications and Adverse Effects

Contraindications to ivabradine encompass bradycardia (e.g., HR < 70 bpm) before treatment initiation, second- and third-degree atrioventricular block, cardiogenic shock, acute myocardial infarction, hypotension (blood pressure less than 90/50 mmHg), sick sinus syndrome, and unstable angina. Additionally, the drug should be avoided in pregnant and lactating women [55].

The most frequently observed adverse events associated with ivabradine include bradycardia, atrial fibrillation, hypertension, and phosphenes. Phosphenes, luminous phenomena in a limited area of the visual field, result from ivabradine interaction with the HCN-1 isoform expressed in retinal photoreceptors, similar to the HCN-4 channels in the sinoatrial node [17,55]. Less commonly reported occurrences encompass rash, diplopia, angioedema, pruritus, urticaria, visual impairment, erythema, and vertigo.

## 4. Ivabradine Use in Cardiovascular Diseases

Ivabradine is prescribed for the symptomatic management of chronic stable angina and ischemic heart disease (IHD), as well as heart failure (HF). It is particularly suitable for individuals who are unable to tolerate β-blockers or have contraindications against their administration.

According to the 2021 European Society of Cardiology (ESC) Guidelines for the management of heart failure and the 2022 American College of Cardiology/American Heart Association and the Heart Failure Society of America (ACA/AHA/HFSA) guidelines [56,57], ivabradine is indicated for symptomatic patients with NYHA class II-IV heart failure with reduced ejection fraction (HFrEF). Specifically, it should be considered as a second-line therapy to reduce the risk of HF hospitalization or cardiovascular death in symptomatic patients with left ventricular ejection fraction (LVEF) ≤ 35%, who are in sinus rhythm with a resting HR ≥ 70 bpm, despite receiving treatment with the maximum tolerated dose of β-blockers, angiotensin-converting-enzyme inhibitors (ACE-I) or angiotensin-receptor blockers (ARB), and mineralocorticoid receptor antagonist (ARNI). In patients who cannot tolerate or have contraindications to β-blockers, ivabradine is recommended as a first-line therapy in combination with ACE-I (or ARB) and ARNI.

In the failing heart, an increase in HR initially compensates for a decrease in stroke volume, to preserve CO. However, prolonged neuroendocrine activation depletes catecholamines in failing myocytes, resulting in hypertrophy, apoptosis, left ventricular remodeling, and a reduction in LVEF [18,58]. In heart failure, the force-frequency relationship becomes inverted: While an increased HR enhances contractile performance (Bowditch effect) in nonfailing myocardium, it leads to a decline in contractile function in failing myocardium, resulting in ventricular-arterial decoupling and alterations of cardiovascular efficiency and cardiac energetic requirements [11,13,18]. Ivabradine acts as an anti-anginal and anti-ischemic agent by reducing HR, thereby decreasing myocardial oxygen consumption, without exhibiting negative inotropic effects or causing coronary vasoconstriction. Ivabradine prolongs diastolic duration, enhancing ventricular filling and coronary blood flow. It also preserves coronary dilation during exercise, increases coronary flow reserve, and improves collateral microcirculatory perfusion. Compared to doubling the dose of β-blockers in patients with HF (and even in comparison to digoxin), ivabradine provides more significant symptomatic relief and results in an increase in left ventricular ejection fraction [17,18,54,59].

## 5. Ivabradine Use in Sepsis

As already reported, during sepsis, tachycardia and the excessive activation of the sympathetic nervous system are compensatory mechanisms to initially preserve CO and DO_2_. However, the persistence of this sympathetic overstimulation can have detrimental effects on myocardial viability, performance, contractility, and its interaction with the arterial vasculature. This can also lead to the emergence of arrhythmogenic foci [20].

Targeting tachycardia and autonomic dysfunction is significantly associated with improved outcomes in critically ill patients [26,33,60]. Ivabradine application in patients with sepsis aims at reducing HR to alleviate myocardial stress. While these patients may require inotropic therapy, the side effects of traditional inotropes, such as vasodilation, increased MVO_2_, myocardial ischemia, and tachyarrhythmias, can limit their use or dosage, potentially blunting their efficacy. In patients with low-output heart failure, elevated cardiac filling pressures, and tissue hypoperfusion, necessitating inotropic support, ivabradine has demonstrated effectiveness in reducing the tachycardic effects of dobutamine, letting its inotropic effects prevail. This minimizes the HR-related adverse effects of dobutamine, resulting in a more efficient cardiac cycle and improved hemodynamics [61,62,63,64,65]. In contrast, β-blockers have been less effective in preventing HR increases in response to dobutamine, potentially attenuating the hemodynamic benefits of this therapy [66].

Human and animal studies have indicated that the use of ivabradine for HR reduction results in a significant decrease in arterial elastance (Ea), a parameter representing left ventricular pulsatile and mean afterload [67,68]. This reduction in overall afterload was primarily attributed to a decrease in vascular pulsatile load, as evidenced by an increase in total arterial compliance, while systemic vascular resistance remained unchanged. This enhancement in total arterial compliance ultimately led to an improved ventricular-arterial coupling, resulting in a substantial increase in stroke volume without affecting left ventricular contractility and cardiac output.

Additionally, there have been reports of the effects of ivabradine on reduced levels of inflammatory cytokines, such as IL-6 and TNF-α, and oxidative stress in animal models of acute heart failure [69,70]. This reduction is presumably linked to the heart rate reduction achieved with ivabradine, which enhances myocardial oxygen supply/consumption balance (thus ameliorating myocardial hypoxia). Furthermore, improved ventricular filling and a subsequent reduction in sympathetic activity may also play a role in diminishing catecholamine-induced cytokine production [69].

In septic shock, lipopolysaccharide (LPS), a key inducer of shock in Gram-negative bacterial sepsis, interacts with HCN channels, leading to both tachycardic and bradycardic effects, with the resultant disturbances in HR and HRV found in septic shock with autonomic dysfunction. Ivabradine I*_f_* blocking potency is preserved in LPS endotoxemia, making it a relevant intervention in these scenarios [28,29,71,72]. In an experimental septic model on hamsters [4], ivabradine also proved effective in ameliorating microvascular abnormalities induced by sepsis because of better organ perfusion due to improved cardiac efficiency. These beneficial effects included increased capillary density, arteriolar diameter, enhanced venular capacitance, and improved venous return. These changes led to a reduction in microcirculatory leakage, resulting in improved vascular retention of resuscitation fluids, reducing the volume of fluids required to achieve a comparable hemodynamic effect. Ultimately, these effects contributed to the enhancement of renal, hepatic, and neurological function. It is worth noting that the I*_f_* current may also be directly stimulated through the nitric oxide (NO)-cyclic guanosine monophosphate (cGMP) pathway, which is known to be upregulated in septic shock and might explain the effects on microcirculation. This pathway represents another potential target for circulatory-focused therapies in the management of septic shock [71].

Despite all these promising findings, clinical data on the use of ivabradine in septic patients remain relatively limited. De Sanctis et al. [5] reported the use of ivabradine in three patients who developed septic shock-related MODS following cardiac surgery. Ivabradine was administered twice daily via a nasogastric tube, with a loading dose of 10 mg followed by a maintenance dose of 5 mg every 12 h for a total of 18 h. In all three patients, HR and cardiac index (CI) decreased, while mean arterial pressure (MAP), stroke volume index (SVI), mixed venous saturation (SvO_2_), and end-diastolic volume index (EDVI) increased. Notably, pulmonary capillary wedge pressure (PCWP) and RAP remained unchanged. Lactate levels, base excess, and organ dysfunction severity improved, while norepinephrine dose requirements were rapidly reduced, ultimately resulting in favorable outcomes for the patients.

In a prospective randomized controlled trial [3], highly selected patients with septic shock and persistent tachycardia (HR > 95 bpm) were administered Ivabradine enterally. The ivabradine group received doses ranging from 2.5 to 3.25 mg every 6 h for a total of 96 h, with a total of 60 patients enrolled, including 30 patients in a standard care control group. In the ivabradine group, both stroke volume index (SVI) and left ventricular ejection fraction (LVEF) were higher, and there were lower requirements for norepinephrine, reduced organ dysfunction severity, lower filling pressures, and decreased serum lactate and N-terminal pro-B-type natriuretic peptide (NT-proBNP) levels. However, there were no significant differences in MAP, CI, or ScvO_2_ between the two groups. Short-term survival at 96 h was notably higher in the Ivabradine group, but there were no significant differences in long-term (30 days) survival or length of stay in the intensive care unit (ICU). The median reduction in HR in the ivabradine group was approximately −26 bpm, which is consistent with the findings from previous studies involving intravenous esmolol [27,73]. This reduction in HR was interpreted as an improvement in cardiac systolic and diastolic performance, resulting in restored ventriculo-arterial coupling, reduced vasopressor requirements, and enhanced lactate clearance due to improved microcirculatory flow.

However, the MODIFY trial [6] presented conflicting results. This trial prospectively involved 70 patients with MODS due to cardiac (coronary subgroup) and/or septic shock (non-coronary subgroup) who were treated with ivabradine at a dose of 5 mg twice daily for 96 h or placebo. While the Ivabradine group showed a trend of improvements in invasive cardiac performance parameters during the 96 h treatment period, the control group experienced further deterioration in hemodynamic variables. Particularly, the ivabradine group had an increase in CI, global end-diastolic volume index (GEDVI), and cardiac power index for both ventricles (LV-CPI, RV-CPI). Notably, according to the subgroup analysis, differences in HR attenuation compared to the control group were observed primarily in the non-coronary MODS group, in patients younger than 70 years old, and in patients with an APACHE II score of ≤35. However, there was no observed attenuation in disease severity between the two groups. This study has several limitations, including a short treatment duration, the use of an arbitrarily predefined heart rate threshold rather than individualized targets, and a heterogeneous patient population with varying degrees of severity and different underlying pathologies. Both the clinical trials from Datta et al. [3] and Nuding et al. [6] had as exclusion criteria patients requiring the use of potent cytochrome P450 3A4 inhibitors, such as antifungals of the azole-type, macrolide antibiotics, and HIV protease inhibitors. As such, the results of these studies are not affected by potential ivabradine pharmacokinetic interactions. Evidence on ivabradine application in human septic shock is thus limited to these 3 studies, including a total of 133 patients. Moreover, therapeutic use was limited In time in all three studies (18 h for De Santis et al. [5]; 96 h for Datta et al. [3]; and Nuding et al. [6]). While hemodynamic parameters were invasively evaluated with transpulmonary thermodilution (either with a pulmonary artery catheter or a PiCCO^®^ system) in the studies from De Santis and Nuding, Datta et al. based their evaluations only on transthoracic echocardiography, with its consequential limitations. Currently, a phase 3 multicenter, prospective, randomized, controlled trial is underway, focusing on ivabradine use in septic shock (NCT04031573).

## 6. Conclusions

Targeting tachycardia and autonomic dysfunction is associated with better outcomes in MODS patients.

**Figure 2:**
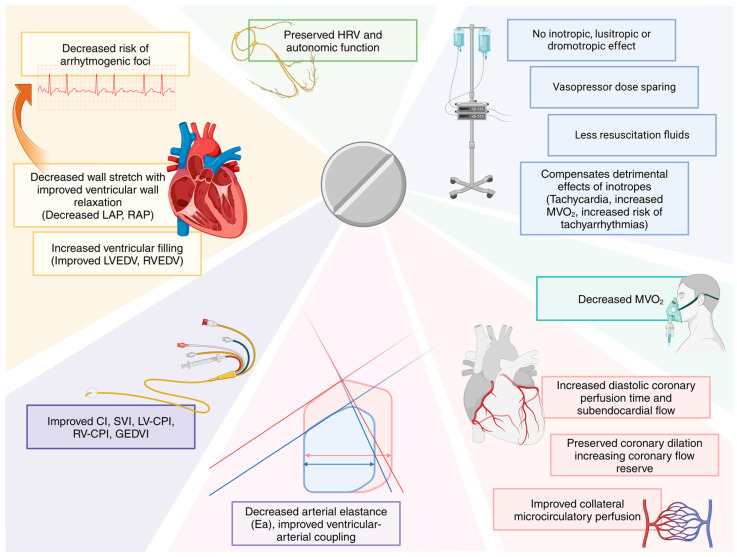
Beneficial effects of heart rate reduction by ivabradine. The ventriculo-arterial coupling graph represents the putative change between tachycardic cardiac dysfunction (blue lines and area) and the ivrabradine effect (red lines and area) on the cardiac cycle and on the coupling between the left ventricle and the arterial elastances. HRV: heart rate variability; MVO_2_: myocardial oxygen consumption; LAP: left atrial pressure; RAP: right atrial pressure; LVEDV: left ventricle end-diastolic volume; RVEDV: right ventricle end-diastolic volume; CI: cardiac index; LV-CPI: left ventricle cardiac power index; RV-CPI: right ventricle cardiac power index; GEDVI: global end-diastolic volume index.

Ivabradine’s selective chronotropic negative effect, in comparison to β-blockers and calcium-channel antagonists, does not exert inotropic, lusitropic, or dromotropic effects and does not impact coronary and peripheral vascular resistances. Although the efficacy of HR reduction is comparable between β-blockers and ivabradine, their mechanisms differ; while the negative inotropic action of β-blockers prolongs both systole and diastole, ivabradine produces a greater prolongation of the diastolic phase of the cardiac cycle, consequently leading to longer coronary perfusion and ventricular filling times when compared to β-blockers. Ivabradine’s overall effect results in a reduction of myocardial oxygen demand, improved ventricular wall relaxation, prolonged coronary perfusion, enhanced subendocardial microvascular perfusion, and improved ventricular end-diastolic volume, with enhanced ventricular-arterial coupling and improved cardiac mechanics, eventually improving the SVI and the CI. Septic shock patients may thus require lower vasopressor and fluid doses, with a resultant adrenergic and fluid overload sparing effect (Figure 2).

Ivabradine emerges as a promising tool for diversifying the treatment of septic shock and sepsis-induced cardiomyopathy, introducing a novel conceptual approach for addressing hemodynamic disturbances in these critically ill patients, a strategy warranting further investigation. Conceptually, the combination of negative chronotropic drugs, such as peripherally acting drugs (e.g., ivabradine, amiodarone, digoxin) with centrally acting chronotropic drugs (e.g., dexmedetomidine, clonidine), could also be potentially investigated, so as to improve sepsis-related cardiac and vasomotor sympathetic derangements, thus improving the final target of every sepsis therapy, namely tissue perfusion.

## Data Availability

Data sharing is not applicable to this article as no new data were created or analyzed in this study.
